# *Clostridium butyricum* and Its Culture Supernatant Alleviate the *Escherichia coli*-Induced Endometritis in Mice

**DOI:** 10.3390/ani12192719

**Published:** 2022-10-10

**Authors:** Cholryong Mun, Jiapei Cai, Xiaoyu Hu, Wenlong Zhang, Naisheng Zhang, Yongguo Cao

**Affiliations:** 1 Department of Clinical Veterinary Medicine, College of Veterinary Medicine, Jilin University, Changchun 130062, China; mirondragon@163.com (C.M.); caijpjlu@163.com (J.C.); huxiaoyu@jlu.edu.cn (X.H.); zwenlong123@126.com (W.Z.); 2 Department of Veterinary Medicine, Pyongsong University of Veterinary Medicine and Animal Husbandry, Pyongsong 950003, Democratic People’s Republic of Korea

**Keywords:** endometritis, *Clostridium butyricum*, culture supernatant, NF-κB signaling pathway, reproductive outcome, *Escherichia coli*

## Abstract

**Simple Summary:**

This study evaluated the therapeutic effect of Clostridium butyricum and its culture supernatant on *Escherichia coli*-induced endometritis in mice. We infused *E. coli* into the uterine cavity of mice and induced endometritis. After 48 hours, *Clostridium butyricum* or its culture supernatant was infused into the uterine cavity of mice. After 48 hours, physiological indicators, uterine morphology, histopathology and uterine bacterial load were examined. The results showed that both *Clostridium butyricum* and its culture supernatant relieved uterine inflammation. In addition, we evaluated the reproductive performance of mice treated with *Clostridium butyricum* and its culture supernatant, and the results showed that they improved the reproductive performance of mice with endometritis. Finally, we have investigated the effect of *Clostridium butyricum* and its culture supernatant on the NF-κB signaling pathway, and the results show that they inhibit the activation of NF-κB signaling pathway. In conclusion, *Clostridium butyricum* and its culture supernatant have a potential therapeutic effect on *Escherichia coli*-induced endometritis.

**Abstract:**

Endometritis is a disease with a high incidence in dairy cows and causes great economic loss to milk production. This study examined the therapeutic effects of *Clostridium butyricum* and its culture supernatant on *Escherichia coli*-induced endometritis in mice. The results showed that *Clostridium butyricum* and its culture supernatant effectively suppressed inflammatory responses of uterine tissues, such as uterine morphological changes, pathological damage, and the production of inflammatory cytokines. *Clostridium butyricum* and its culture supernatant significantly decreased uterine microbial loads. In addition, *Clostridium butyricum* and its culture supernatant restored reproduction outcomes in *Escherichia coli*-induced endometritis mice. Western blot analysis showed that *Clostridium butyricum* and its culture supernatant suppressed the NF-κB signaling pathway. Therefore, the anti-inflammatory mechanism of *Clostridium butyricum* and its culture supernatant may occur through the anti-bacterial activity and regulation of the expression of NF-κB in the uterus. The anti-inflammatory effect of the culture supernatant of *C. butyricum* was slightly better than that of viable *C. butyricum*. Therefore, our experimental results showed that *Clostridium butyricum* culture supernatant may be an effective drug for treating endometritis.

## 1. Introduction

Endometritis is an infectious disease that occurs frequently in humans and animals and is an important disease that causes a decrease in fertility and consequently a decrease in milk productivity, especially in cows [1,2]. *Escherichia coli* (*E. coli*), *Trueperella pyogenes*, *Fusobacterium necrophorum*, *Prevotella*, and *Bacteroides* species are the primary pathogens isolated from the uterus of cows with endometritis [3,4,5,6]. In particular, *E. coli* is one of the most common pathogens in the uterine secretion of cows with clinical endometritis [7]. *E. coli* stimulates endometrial epithelial cells to produce inflammatory cytokines such as TNF-α, IL-1β, and IL-6 and destroys the tight junction between epithelial cells, resulting in inflammation of the uterine tissue [8]. The common treatment methods for endometritis currently used in clinical practice are systemic antibiotics and hormone therapy, and in severe cases, drug disinfection of the uterus is used. Using antibiotics has a long history and has a relatively good therapeutic effect. Clinically, antibiotics such as ceftiofur, cephapirin, and ampicillin are used in the treatment of endometritis in dairy cows. Ampicillin is a β-lactam antibiotic that inhibits the synthesis of peptidoglycan and other components of the bacterial cell wall [9]. Ampicillin received approval in 1998 for use in dairy cattle in the United States (Center for Veterinary Medicine, Food and Drug Administration new animal drug application 200–180; FDA, 1998), and it is indicated for the therapy of infections caused by *E. coli* [10,11]. Intramuscular injection of ampicillin at a dose of 11 mg/kg body weight once a day achieved therapeutic concentrations in the milk, lochial fluid, and endometrial tissue of postpartum dairy cows [12]. Intrauterine infusion of the drug is also a recommended treatment for uterine diseases. It has been reported that intrauterine treatment of ampicillin and cloxacillin plus intramuscular injection of ampicillin resulted in a good therapeutic effect [13]. Although the use of antibiotics has a long history, this currently limits its use because of problems such as the emergence of resistant bacteria and food safety [14]. Hormone therapy is an adjuvant treatment method that is effective for uterine involution after delivery. Representative hormone agents are oxytocin, estrogen, and prostaglandin [15].

Probiotics are symbiotic microorganisms that have beneficial effects on the host’s life activities, such as activating the immune system [16] and anti-inflammatory action [17]. Recently, some studies have reported the therapeutic effect of probiotics on endometritis in dairy cows. For example, a researcher reported that lactic acid bacteria reduced metritis prevalence and inflammation of the bovine endometrium [18,19].

*Clostridium butyricum* (*C. butyricum*) is a Gram-positive anaerobic rod bacterium that ferments carbohydrates such as glucose, sucrose, fructose, and starch to synthesize organic acids such as butyric acid, acetic acid, and lactic acid [20]. *C. butyricum* exhibits anti-pathogenic activity in the digestive tract of humans and animals. It has beneficial activity against other beneficial bacteria [21]. In addition, this bacterium exerts various beneficial effects such as intestinal epithelial cell protective activity [22] and anti-inflammatory effects [23]. Butyric acid, the most important metabolite of this bacterium, also exhibits various beneficial effects such as intestinal epithelial protection, anti-inflammatory effects, and anticancer effects [24]. However, the effect of *C. butyricum* on endometritis has not been reported yet. Thus, the present study investigated the potential protective effects and mechanisms of *C. butyricum* on *E. coli*-induced endometritis.

## 2. Materials and Methods

### 2.1. Animals

Eight-week-old female BALB/c mice (22–25 g) were purchased from Liaoning Changsheng Biotechnology Co., Ltd. (Benxi, China). The mice were housed in a room with a temperature of 24 ± 1 °C and a relative humidity of 40–80%. Food and water were supplied ad libitum. All animal procedures were performed under the Guidelines for Care and Use of Laboratory Animals of Jilin University, and the experiments were approved by the Animal Ethics Committee of Jilin University. 

### 2.2. Materials

*Escherichia coli* MTCC 1652 were obtained from Inner Mongolia Agricultural University, China, and cultured at 37 °C in LB broth with continuous shaking. *Clostridium butyricum* BNCC 337239 was purchased from BeNa Culture Collection (BNCC), Kunshan, China, and cultured at 37 °C in a reinforced clostridial medium with an anaerobic condition. Mouse monoclonal antibodies, NF-κB p-p65, and p-IκBα were purchased from Cell Signaling Technology Inc. (Danvers, MA, USA).

### 2.3. Preparation of the Culture Supernatants of C. butyricum

*C. butyricum* was cultured in a reinforced clostridial medium (RCM) under an anaerobic condition for 24 h at 37 °C. The bacterial concentration of this culture broth is 1 × 10^9^ CFU/mL. The culture broth was centrifuged at 5000× *g* for 10 min at 4 °C, and the supernatants were filtered through a 0.2-μm pore size syringe filter. This supernatant is defined as a high-concentration culture supernatant of *C. butyricum* (HSC), and the solution diluted 10 or 20 times with physiological saline is defined as the medium-concentration culture supernatant (MSC) and low-concentration culture supernatant of *C. butyricum* (LSC), respectively.

### 2.4. Uterine Infusion

All agents were infused through the vagina into the uterine cavity. We used a yellow pipette tip and blunt needle for uterine infusion. The tip of the yellow pipette was slightly clipped off, and this was slowly inserted into the mouse’s vagina until the tip reached the cervix. The 23-gauge blunt needle (50 mm length) of the micro syringe (0.1 mL) was then passed through the yellow pipette tip and the cervix into the uterine cavities.

### 2.5. Experimental Design

**Figure 1:**
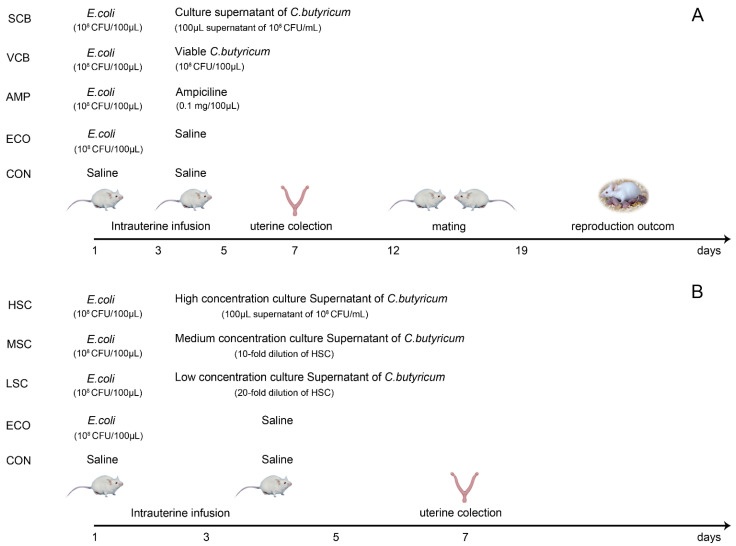
Timeline of animal experiments. (**A**) Experimental design of the effect of *C. butyricum* and its culture supernatant on *E. coli*-induced endometritis in mice. (**B**) Experimental design of the effect of different concentrations of *C. butyricum* culture supernatant on *E. coli*-induced endometritis in mice.

To explore the effect of *C. butyricum* and its culture supernatant in repairing uterine inflammatory injury, 60 female BALB/c mice were randomly divided into five groups: control group (CON), *E. coli* group (ECO), ampicillin group (AMP), viable *C. butyricum* group (VCB), and supernatant of *C. butyricum* group (SCB). For comparison with antibiotic treatment, we included ampicillin as the antibiotic treatment control group. Drillich, M. et al. reported that the uterine infusion concentration of ampicillin in cattle is 2500 mg/cattle [13]. Therefore, according to the drug dose ratio of body weight, 0.1 mg of ampicillin was infused into the uterine cavity of each mouse (A cow weighs about 500 kg, and a mouse weighs about 20 g; thus, the weight ratio is about 25,000:1). On day 1, mice in the CON group were infused with 100 μL of saline, and mice in the other groups were infused with 100 μL of 1 × 10^9^ CFU/mL E. coli. On days 3 and 5, mice in the CON and ECO groups were infused with 100 μL of saline, mice in the AMP group were infused with 100 μL ampicillin (1 mg/mL), mice in the VCB group were infused with 100 μL of 1 × 10^8^ CFU/mL *C. butyricum*, and mice in the SCB group were infused with 100 μL of culture supernatant of *C. butyricum* (1 × 10^8^ CFU/mL). All agents were infused through the vagina into the uterine cavity. On day 7, 6 mice from each group were euthanized, the uterus was collected, and the remaining mice could stay together with the male for 1 week from day 12 to 18; then, the reproduction outcome was evaluated (Figure 1A).

To examine the anti-inflammatory effect of bacterial culture supernatant on endometritis according to the concentration, 40 female BALB/c mice were randomly divided into five groups: control group (CON), *E. coli* group (ECO), low concentration supernatant of *C. butyricum* group (LSC), medium concentration supernatant of *C. butyricum* group (MSC), and high concentration supernatant of *C. butyricum* group (HSC). On day 1, the CON group was infused with 100 μL of saline, and the other groups were infused with 100 μL of 1 × 10^9^ CFU/mL *E. coli*. On day 3 and 5, CON and ECO groups were infused with 100 μL of saline, and LSC, MSC, and HSC groups were infused with 100 μL of corresponding culture supernatant of *C. butyricum*. On day 7, all mice were euthanized, and the uterus was collected (Figure 1B).

### 2.6. Body Temperature and Weight Measurement

The body temperature of each mouse was measured rectally on day 1 and day 7 with the aid of a lubricated digital probe thermometer (model Panlab-0331, Beijing, China). The body weights of the animals were recorded on day 1 and day 7 (before sampling).

### 2.7. Histopathological Examination of the Uterine Tissues

Collected uterine tissues from each group were kept in 4% paraformaldehyde for 48 h. The samples were embedded in paraffin and cut into 4 μm slices. After dewaxing, the sections were stained with hematoxylin and eosin for histological assessment under a light microscope. The histopathologic scoring method of the uterine tissue is listed in Table S1, according to the modification of a previously reported method [25]. The major histopathological indicators were evaluated by endometrial injury, inflammatory infiltrate, uterine edema, and endometrium thickness (graded 0–3, from normal to severe, including normal, mild, moderate, and severe).

### 2.8. Uterine Bacterial Loads Examination

To determine the bacterial load in the uterus, the uterine cavity was lavaged with 1 mL of sterile saline using a micro syringe. Serial log dilutions were made, and 100 microliters of each dilution were then plated on an LB plate and incubated at 37 °C for 24 h under aerobic conditions. Colony-forming units were counted. Results were expressed as colony-forming units per uterus.

### 2.9. ELISA Assay

The uterine tissues were prepared and homogenized with cold PBS (weight/volume ratio 1:9) on ice. The homogenates were centrifuged at 2000× *g* for 40 min at 4 °C. The levels of pro-inflammatory cytokines TNF-α and IL-1β in supernatants were detected using ELISA kits (Biolegend, San Diego, CA, USA) according to the manufacturer’s instructions. The read absorbance of the samples was tested at 450 nm using a microplate reader.

### 2.10. Evaluation of Mice Reproductive Outcomes

Reproductive outcomes were evaluated using three indicators. The pregnancy rate refers to the percentage of pregnant mice to the number of mated mice in each group. Pups per litter refers to the total number of pups born in each group divided by the number of maternal mice in the group. Weight per pup is the total weight of pups in each group divided by the number of pups in that group.

### 2.11. Western Blotting Assay

The total proteins were extracted from the uterine tissues using a tissue protein extraction reagent (T-PER). The protein concentration was determined by using a BCA protein assay kit. A total of 40 μg of protein was fractionated using 10% SDS-PAGE and transferred onto polyvinylidene difluoride (PVDF) membranes. Subsequently, the membranes were blocked with 5% skimmed milk powder for 2 h at room temperature on a shaker, followed by overnight incubation at 4 °C with the specific primary antibodies and were washed with TBS-T three times. After incubation with the horseradish peroxidase (HRP)-conjugated secondary antibody for 45 min at room temperature, the membranes were washed with TBS-T another three times and incubated with enhanced chemical luminescence detection solution to detect the intensities of the proteins using an ECL Plus western blotting detection system. β-actin protein served as an internal control.

### 2.12. Statistical Analysis

Data in the present study were analyzed by GraphPad prism 9. All values are expressed as the means ± SEM. The difference between the mean values of normally distributed data was analyzed using one-way ANOVA (Dunnett’s *t*-test) and two-tailed Student’s *t*-test. *p* < 0.05 was used as the criterion for statistical significance.

## 3. Results

### 3.1. Body Temperature and Body Weight Analysis

No significant differences were observed in the body temperature of the different groups (Table S1). However, there was a slight decrease in the ECO group (Table S2). There was a statistically significant difference in body weight between day 1 and day 7 in all experimental groups, except the healthy group (Figure S1).

### 3.2. C. butyricum and Its Culture Supernatant Alleviated Inflammatory Response of the Uterine Tissues Induced by E. coli

**Figure 2:**
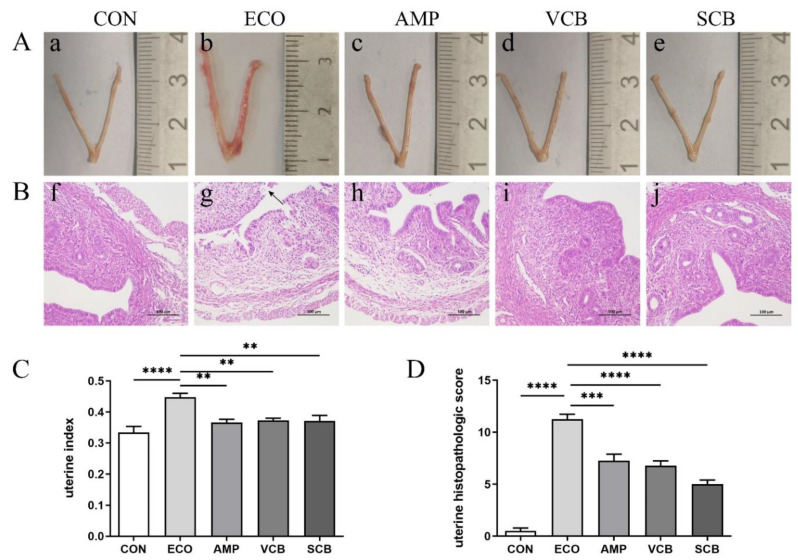
*C. butyricum* and its culture supernatant alleviated uterine morphological and histopathological changes induced by *E. coli*. (**A**) Uterus morphology ((**a**) CON; (**b**) ECO; (**c**) AMP; (**d**) VCB; (**e**) SCB). (**B**) H&E staining of uterine tissue ((**f**) CON; (**g**) ECO; (**h**) AMP; (**i**) VCB; (**j**) SCB). (**C**) Uterine index. (**D**) Histopathologic scoring of the uterine tissue. The values presented are the means ± SEM (*n* = 6). ** *p* < 0.01, *** *p* < 0.001, and **** *p* < 0.0001 are significantly different from ECO.

Uterine morphology, H&E staining, and uterine index were used to evaluate the protective effect of *C. butyricum* and its culture supernatant on endometritis induced by *E. coli* in mice. On the morphological characteristics of the uterus, severe erythema was seen in the ECO group but not in other groups (Figure 2A). In H&E staining, the ECO group showed severe destruction of the endometrial epithelial layer and neutrophil infiltration, but the other groups had significant remission (Figure 2B). In addition, the uterine histopathological score also indicated that treatment groups alleviated uterine tissue damage induced by *E. coli.* In addition, the histopathological scores of the VCB group were lower than those of the AMP and SCB groups (Figure 2D). In the uterine index, the ECO group had the highest level, and the three treatment groups (AMP, VCB, and SCB) recovered remarkably (Figure 2C).

### 3.3. C. butyricum and Its Culture Supernatant Alleviated Pro-Inflammatory Cytokines of the Uterine Tissues Induced by E. coli

**Figure 3:**
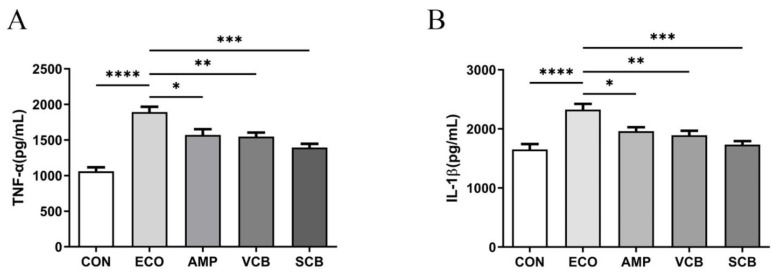
*C. butyricum* and its culture supernatant reduced the production of pro-inflammatory cytokines in uterine tissues induced by *E. coli*. (**A**) Uterine tissue TNF-α. (**B**) Uterine tissue IL-1β. The values presented are the means ± SEM (*n* = 6). * *p* < 0.05, ** *p* < 0.01, *** *p* < 0.001, and **** *p* < 0.0001 are significantly different from the ECO group.

TNF-α and IL-1β are both representative cytokines of inflammation. The results show that the production of TNF-α and IL-1β in the uterine tissues of the VCB and SCB groups was significantly lower than that of the ECO group. The SCB group had slightly lower levels of inflammatory cytokines than the VCB group (Figure 3).

### 3.4. C. butyricum and Its Culture Supernatant Reduced the Bacterial Load of the Mouse Uterus

**Figure 4:**
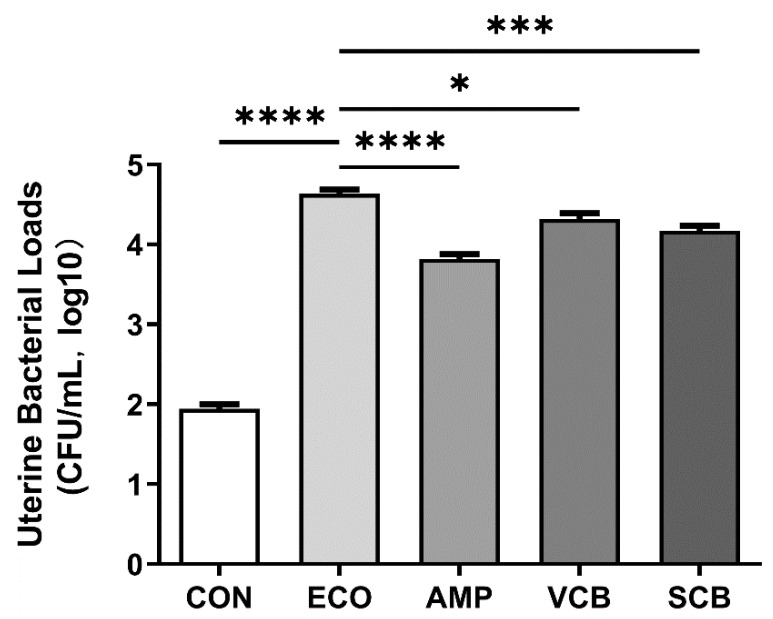
*C. butyricum* and its culture supernatant reduced the uterine bacterial loads. The values presented are the means ± SEM (*n* = 6). * *p* < 0.05, *** *p* < 0.001, and **** *p* < 0.0001 are significantly different from ECO group.

We evaluated the effect of *Clostridium butyricum* and its culture supernatant on intrauterine bacterial load. The intrauterine bacterial load of the AMP group was significantly lower than that of the ECO group, and the bacterial load of VCB and SCB groups also decreased significantly (Figure 4).

### 3.5. C. butyricum and Its Culture Supernatant Restore Reproduction Outcome in E. coli-Induced Endometritis Mice

**Figure 5:**
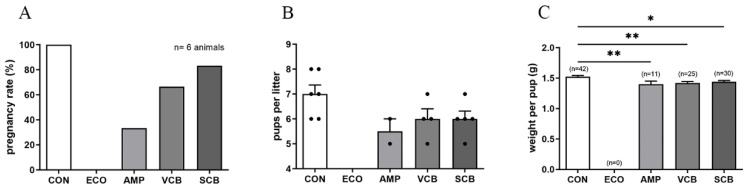
*C. butyricum* and its culture supernatant restore reproduction outcome in *E. coli*-induced endometritis mice. (**A**) Pregnancy rate refers to the percentage of the number of pregnant mice to the number of mated mice in each group (the number of mated mice in each group = 6 animals). (**B**) Pups per litter refers to the total number of pups born in each group divided by the number of maternal mice in the group. The number of black dots indicates the number of pups. (**C**) Weight per pup is the total weight of pups in each group divided by the number of pups in that group. * *p* < 0.05 and ** *p* < 0.01 are significantly different from the CON group.

The reproduction outcome indexes, including pregnancy rate, pups per litter, and weight per pup, were measured after giving birth. None of the CON became pregnant. Pregnancy rates in the VCB and SCB groups were higher than in the AMP group but were lower than in the CON group. In addition, the pregnancy rate in the SCB group was higher than that in the VCB group (Figure 5A and Table S3). The pups per litter significantly decreased in all treatment groups compared to the CON group, but there was no statistical difference between the VCB and SCB groups (Figure 5B and Table S4). The weight per pup in the treatment groups was lower than that in the CON group; however, there was no statistical difference between the AMP, VCB, and SCB groups (Figure 5C and Table S5).

### 3.6. C. butyricum Culture Supernatant Attenuates E. coli-Induced Endometritis in a Concentration-Dependent Manner

**Figure 6:**
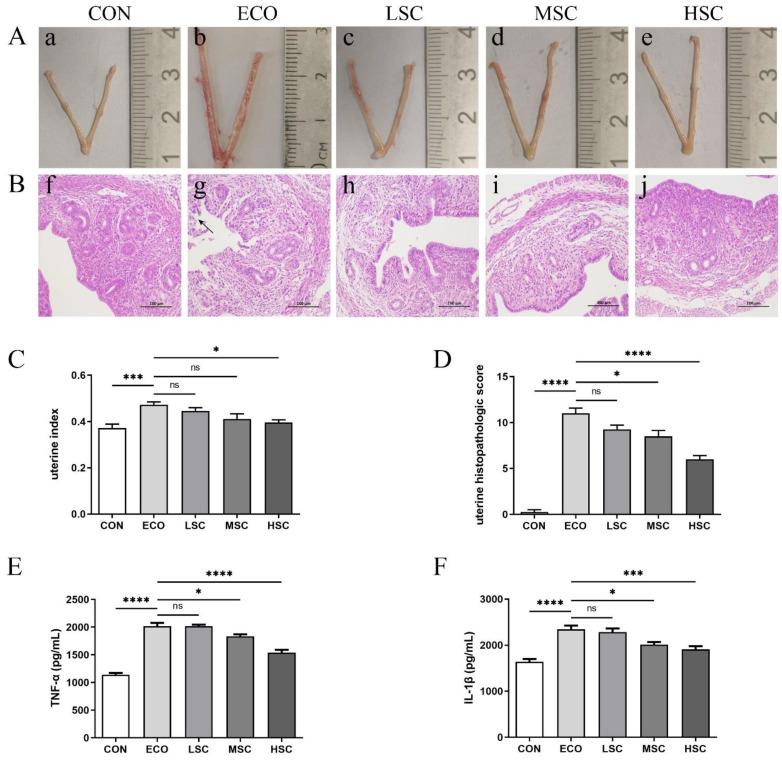
*C. butyricum* culture supernatant relieved the uterine tissue inflammatory response induced by *E. coli* in a concentration-dependent manner. (**A**) Mouse uterus morphology ((**a**) CON; (**b**) ECO; (**c**) LSC; (**d**) MSC; (**e**) HSC). (**B**) H&E staining of uterine tissue ((**f**) CON; (**g**) ECO; (**h**) LSC; (**i**) MSC; (**j**) HSC). (**C**) Uterine index. (**D**) Histopathologic scoring of the uterine tissue. (**E**) Uterine tissue TNF-α. (**F**) Uterine tissue IL-1β. The values presented are the means ± SEM (*n* = 3). * *p* < 0.05, *** *p* < 0.001, and **** *p* < 0.0001 are significantly different from ECO group.

We tested the effect of different concentrations, including low (LSC), medium (MSC), and high (HSC) concentrations of *C. butyricum* culture supernatant, on endometritis in mice. In the uterine morphology photographs, the uterus of the ECO group had severe erythema, and the uterus of the LSC and MSC groups also had local erythema (Figure 6A). There was no difference in uterine index between the ECO, LSC, and MSC groups, but the HSC group was lower than the ECO group (Figure 6C). H&E staining (Figure 6B) and the uterine histopathological score (Figure 6D) in the treatment groups were dose-dependently alleviated according to the concentration of the *C. butyricum* culture supernatant.

### 3.7. C. butyricum and Its Culture Supernatant Inhibited the Activation of the NF-κB Signaling Pathway Induced by E. coli

**Figure 7:**
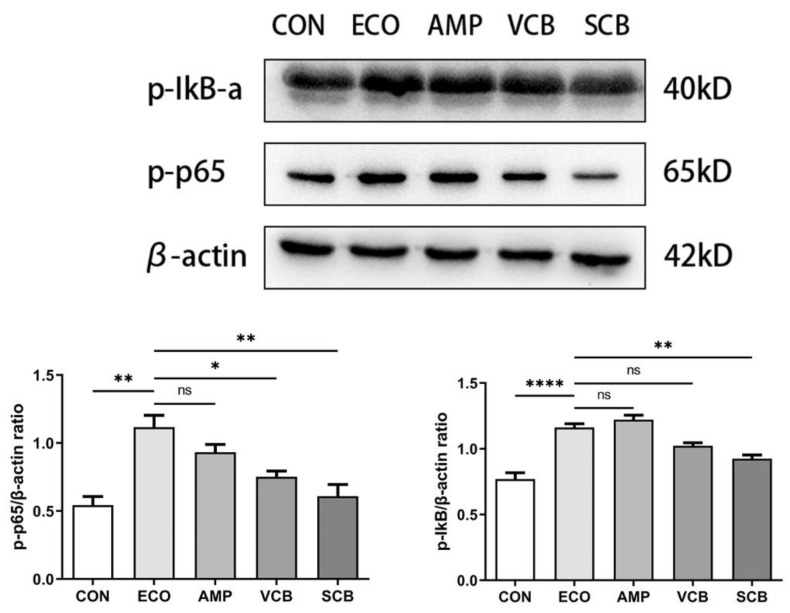
The supernatant of *C. butyricum* inhibited the activation of the NF-κB signaling pathway induced by *E. coli*. Western blot was used to measure p-p65 and p-IκB proteins in uterine tissues. β-Actin was used as a control. The values presented are the means ± SEM (*n* = 3). * *p* < 0.05, ** *p* < 0.01 and **** *p* < 0.0001 are significantly different from ECO group.

We examined the effect of the *C. butyricum* and its culture supernatant on the NF-κB signaling pathway. The results showed that *C. butyricum* and its culture supernatant inhibited the expression of the p-p65 and p-IκB proteins induced by *E. coli* in uterine tissues (Figure 7).

## 4. Discussion

Endometritis is a common postpartum disease and has been causing great economic loss, especially in farming dairy cows. Probiotics have already been widely used in the prevention and treatment of diseases in humans and animals [26,27]. Its effect is not only in digestive tract diseases but also in the prevention and treatment of many diseases such as diabetes [28]. Several studies have reported that the uterine infusion or vaginal infusion of probiotics is effective in the treatment of endometritis [29,30,31]. *C. butyricum* has been shown to have potential protective or ameliorating effects on a variety of human and animal diseases, including gut-acquired infections, irritable bowel syndrome, inflammatory bowel disease, neurodegenerative disease, metabolic disease, and colonic rectal cancer [23,32]. However, the effect of *C. butyricum* on endometritis has not been reported.

In this study, we examined the therapeutic effect of the probiotic *C. butyricum* and its culture supernatant on *E. coli*-induced mouse endometritis. Endometritis is mainly caused by postpartum pathogen infection, and one of the main pathogens is *E. coli* [8,19]. Therefore, in this experiment, *E. coli* was used to cause endometritis. Antibiotics are currently the most widely used clinical treatment for endometritis. It has been reported that cows receiving an intramuscular injection of 15 mg/kg body weight amoxicillin trihydrate plus the intrauterine infusion of 8000 mg oxytetracycline dihydrate (200 mg/mL) showed good therapeutic results [33]. Another study reported that the intrauterine treatment of ampicillin 2500 mg and cloxacillin 2500 mg plus intramuscular injection of ampicillin 6000 mg for three consecutive days resulted in a good therapeutic effect [13]. We also evaluated the sensitivity of the *E. coli* used in the modeling process to ampicillin and confirmed that it was very sensitive to ampicillin. Therefore, we used ampicillin as the positive treatment control. The experimental results showed that the gross changes of the uterus (erythema) and histological damage of the endometrium (destruction of the endometrial epithelial layer, infiltration of neutrophils) caused by *E. coli* were all alleviated in the treatment groups. However, the treatment effect of the AMP group is not as good as that of VCB and SCB groups. The uterine index was also significantly lower in the treatment groups compared to the ECO group, but there was no difference between the AMP and VCB or SCB groups. TNF-α and IL-1β content increases in inflamed tissues, including endometritis [34]. These inflammatory cytokines were significantly lowered in VCB and SCB groups. The intrauterine bacterial load is one of the important indicators used to evaluate the severity of endometritis [35]. To examine the antibacterial effect of *C. butyricum* and its culture supernatant, the intrauterine bacterial load was measured. The results showed that the uterine bacterial load was significantly reduced not only in the AMP group but also in the VCB and SCB groups. This indicated that *C.* *butyricum* and its culture supernatant had an antibacterial effect. The antibacterial effect of *C. butyricum* has been reported in several studies [32,36]. Our results once again proved the data of previous studies.

The negative effect of endometritis in cows is that it causes infertility and therefore a decrease in milk production. Therefore, the improvement of fecundity after the treatment of this disease is an important index to evaluate the therapeutic effect of endometritis. According to previous studies, the use of antibiotics alleviates the clinical symptoms of endometritis to a certain extent, but there is no good effect on restoring reproduction outcomes [37]. Thus, we tested whether the *C. butyricum* and its culture supernatant would be effective in restoring reproduction outcomes such as pregnancy rate, pups per litter, and weight per pup. The results showed that these reproductive outcome indicators in the *C. butyricum* and its culture supernatant treatment groups were higher than those in the ampicillin-treated group. The mechanism of *C. butyricum* and its culture supernatant improving the reproductive performance of mice with endometritis needs to be further studied.

Through the above experiment, we found that the treatment effect of *C. butyricum* culture supernatant on endometritis was slightly better than that of live bacteria. We speculated that the reason is as follows. The culture supernatant of *C. butyricum* contains abundant antibacterial and anti-inflammatory substances such as short-chain fatty acids and other metabolites. Butyric acid inhibits pathogens and improves intestine barrier function [38], benefits intestinal microbiota, and stimulates immune factors [39,40]. However, the growth activity of *C. butyricum* in utero was relatively weak compared with that of the medium; thus, no sufficient effective substances were synthesized in the short time between the infusion of bacterial suspension and the collection of uteri.

Since the culture supernatant has a better effect than live bacteria and it is more convenient to use culture supernatant than live bacteria in clinical application, we evaluated the therapeutic effect of different concentrations of culture supernatant. The results showed that the *C. butyricum* culture supernatant exhibits a therapeutic effect in a concentration-dependent manner.

Subsequently, we conducted an experiment to explain the treatment mechanism of *C. butyricum* and its culture supernatant for *E. coli*-induced endometritis. One of the major mechanisms of *E. coli*-induced endometritis is related to the activation of the NF-κB pathway by LPS, a major pathogenic factor [41]. NF-κB is an important transcription factor that is localized to the cytosol and bound to its inhibitor IκBα in an inactive state. The NF-κB p65 unit is dissociated and phosphorylated from IκBα by an inducer such as bacterial LPS. The phosphorylated NF-κB is translocated into the nucleus, resulting in the activation of the NF-κB-regulated target genes, such as TNF-α and IL-1β [42,43]. Our result showed that *C. butyricum* and its culture supernatant significantly inhibited the *E. coli*-induced activation of the NF-κB pathway in the uterine tissue.

Although this study indicated that *C. butyricum* and its culture supernatant have therapeutic effects on *E. coli*-induced endometritis in mice, there is a great difference between mouse endometritis and cow endometritis; thus, further experiments in cows are needed.

## 5. Conclusions

*C. butyricum* and its culture supernatant alleviated *E. coli*-induced endometritis and restored the reproduction outcomes of the uterus in mice. The therapeutic mechanism of *C. butyricum* and its culture supernatant on endometritis may be related to the inhibition of *E. coli* growth and the blocking of the NF-κB signaling pathway.

## Data Availability

The datasets generated and/or analyzed during the current study are available from the corresponding author on reasonable request.

## Supplementary Materials

The following supporting information can be downloaded at: https://www.mdpi.com/2076-2615/12/19/2719/s1, Figure S1: Comparison of body weight of mice before and after the experiment; Table S1: The score criterion of uterine injury score; Table S2: Body temperature; Table S3: Pregnancy rate; Table S4: Pups per litter; Table S5: Weight per pup.
