# Supplementary material for: Clostridium butyricum and Its Culture Supernatant Alleviate the Escherichia coli-Induced Endometritis in Mice
Source: Animals (Basel). 2022 Oct 10;12(19):2719. doi: 10.3390/ani12192719 (PMC9559394; doi:10.3390/ani12192719)
Supplement: Supplementary file 1 [file animals-12-02719-s001.zip › animals-1946959-supplementary.pdf]

Table S1. The score criterion of uterine injury score

Endometrial injury		Inflammatory infiltrate		Uterine oedema		Endometrium thickness	
Grade	Score	Grade	Score	Grade	Score	Grade	Score
Normal	0	Normal	0	Normal	0	Normal	0
+	1	+	1	+	1	+	1
++	2	++	2	++	2	++	2
+++	3	+++	3	+++	3	+++	3
Note: "+" was represented as the degree of uterine injury.							

Table S2. Body temperature

Individual number	CON		ECO		AMP		VCB		SCB	
	day 1	day 6	day 1	day 6	day 1	day 6	day 1	day 6	day 1	day 6
1	37.9	37.5	38.6	37.4	37.7	37.5	38	37.7	38.3	38.2
2	38.6	38.8	38.2	36.5	38.2	38.3	38.1	38.4	38.5	38.4
3	38.4	38.6	38.1	36.8	38.4	38.2	38.5	38.8	38.2	38.7
4	38.7	38.5	37.8	35.5	38.7	38.1	38.6	37.9	38.6	38
5	38.3	38.7	38.5	35.6	38.3	38.7	38.2	38.7	37.8	38.1
6	38.1	38.3	38.4	37.1	37.9	38.2	37.8	37.5	38.4	37.7

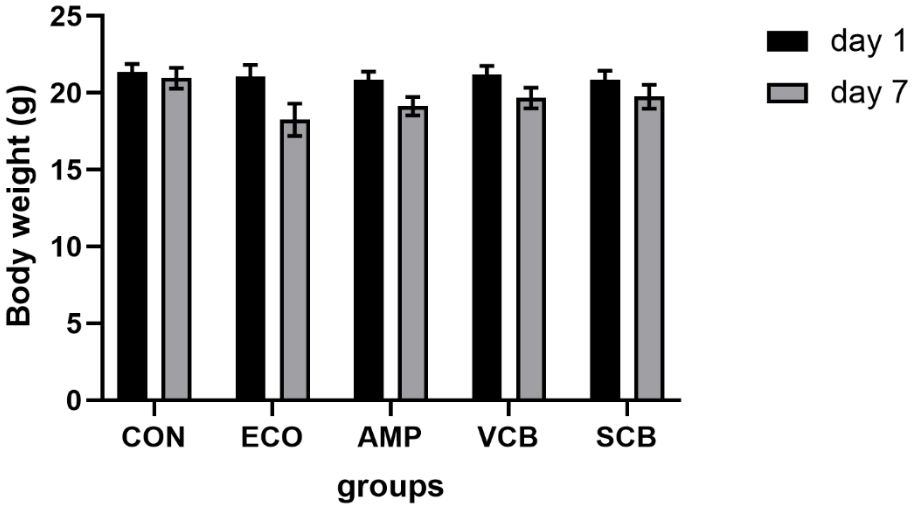

Figure S1. Comparison of body weight of mice before and after the experiment.

Table S3. Pregnancy rate

group	CON	ECO	AMP	VCB	SCB
Total number of female mice	6	6	6	6	6
Number of pregnant female mice	6	0	2	4	5
pregnancy rate (%)	100	0	33.3	66.6	83.3

Table S4. Pups per litter

group	CON	ECO	AMP	VCB	SCB
pups per litter	6		6	5	6
	7		5	7	6
	6			6	6
	8			6	7
	8				5
	7				

Table S5. Weight per pup

group	CON	ECO	AMP	VCB	SCB
	1.6		1.4	1.4	1.3
	1.4		1.6	1.65	1.4
	1.5		1	1.44	1.5
	1.7		1.5	1.33	1.4
	1.5		1.6	1.33	1.3
	1.6		1.4	1.53	1.4
	1.6		1.4	1.2	1.5
	1.7		1.3	1.6	1.3
	1.5		1.5	1.2	1.5
	1.5		1.3	1.3	1.5
	1.5		1.4	1.5	1.5
	1.4			1.4	1.6
	1.6			1.6	1.5
	1.6			1.5	1.5
	1.7			1.5	1.3
	1.5			1.4	1.2
	1.4			1.3	1.5
	1.6			1.4	1.4
	1.5			1.6	1.5
	1.5			1.4	1.6
weight of pup (g)	1.6			1.4	1.4
	1.4			1.3	1.6
	1.7			1.5	1.4
	1.5			1.4	1.5
	1.6			1.5	1.2
	1.6				1.5
	1.5				1.4
	1.5				1.6
	1.6				1.4
	1.4				1.5
	1.3				
	1.5				
	1.6				
	1.3				
	1.5				
	1.7				
	1.6				
	1.5				
	1.6				
	1.3				
	1.2				

	1.6				
